# CIT tumor lines: A series of immunogenic murine cutaneous squamous cell carcinoma cell lines derived from chemical carcinogenesis

**DOI:** 10.1016/j.xjidi.2026.100477

**Published:** 2026-04-15

**Authors:** Robert Letchworth, Miho Tanaka, Alina A. Barnes, Lotus Lum, Savannah Hughes, Grant Schlauderaff, Piyush Chaudhary, Kenneth M. Ng, Daphne Superville, Jadyn Leek, Joshua Tay, Corinna Martinez Luna, Maria Gonzalez, Eric Smith, Dekker C. Deacon, Allie Grossmann, Melissa Q. Reeves

**Affiliations:** 1 Huntsman Cancer Institute, University of Utah, USA; 2 Department of Pathology, University of Utah, USA; 3 Department of Microbiology and Immunology, University of California, San Francisco, USA; 4 Department of Dermatology, University of Utah, USA; 5 Earle A. Chiles Research Institute, Providence Cancer Institute of Oregon, Portland, Oregon, USA

**Keywords:** Cutaneous squamous cell carcinoma, Immune checkpoint inhibitor, Mouse models of cancer, Tumor immunology, Tumor neoantigen

## Abstract

Immunotherapy is widely used to treat advanced-stage skin cancer, but it is effective for only approximately half of patients with skin cancer. To overcome current barriers, preclinical mouse models that faithfully recapitulate the genetics, mutation burdens, and neoantigen patterns of specific human tumor types are essential. However, while many models exist for melanoma, there are few syngeneic murine models of cutaneous squamous cell carcinoma, which is responsible for nearly as many deaths as melanoma each year. Here, we describe a series of 11 cutaneous squamous cell carcinoma tumor lines, the carcinogen-induced tumor (CIT) lines, syngeneic to the FVB strain, that address this need. The CIT lines were established from skin carcinomas induced by 7,12-dimethylbenzanthracene and 12-O-tetradecanoylphorbol-13-acetate treatment and harbor genetic drivers and mutational burdens that recapitulate key features of cutaneous squamous cell carcinoma. Each CIT line gives rise to tumors with a consistent immune infiltration pattern, ranging from T cell-rich “hot” tumors to T cell-poor “cold” tumors. Hot CIT lines exhibit partial responses to immune checkpoint inhibitors, and we have identified two neoantigens present in an immunotherapy-responsive CIT line. The CIT lines thus provide a valuable series of preclinical models for studying anti-tumor immune responses and developing strategies to improve immunotherapy efficacy in cutaneous squamous cell carcinoma.

## Introduction

Cutaneous squamous cell carcinoma (cSCC) is the second most common cancer type in the United States, affecting approximately 700,000 people annually (Karia et al, 2013; Nehal and Bichakjian, 2018; Rogers et al, 2015) and responsible for between 8000 and 15,000 deaths per year (Karia et al, 2013; Mansouri and Housewright, 2017). Although cSCC carries a relatively low risk of local advancement or distant metastasis, its high incidence rate means that as many or more Americans are estimated to die every year from cSCC as from melanoma (Mansouri and Housewright, 2017). Among high-risk patients with cSCC, up to 5% will progress to locally advanced or metastatic tumors that cannot be treated with surgery or radiation alone (Brantsch et al, 2008; Karia et al, 2013; Mourouzis et al, 2009). Immune checkpoint inhibitors (ICIs), which have revolutionized cancer treatment over the past decade (Chen and Mellman, 2017; Ribas and Wolchok, 2018), are approved for advanced cSCC and hold immense potential to be curative for these patients (Ansary et al, 2022; Gross et al, 2022). However, overall response rates of cSCC to ICI therapy are only 50–60% in locally advanced disease (Gross et al, 2022; Hughes et al, 2021; Migden et al, 2018) and drop as low as 35% in metastatic disease (Hughes et al, 2021). These statistics highlight an unmet clinical need to understand why ICI fails for many patients with cSCC and how immunotherapy strategies can be improved for these patients (Grob et al, 2020; Gross et al, 2022; Hughes et al, 2021; Migden et al, 2018).

Murine models play a vital role in basic and translational cancer research, providing a means of conducting mechanistic experiments to dissect cancer and immune cell biology and their interactions with therapy. While autochthonous models, such as the 7,12-dimethylbenzanthracene/12-O-tetradecanoylphorbol-13-acetate (DMBA/TPA) carcinogenesis model for cSCC (Abel et al, 2009; Quintanilla et al, 1986), have made significant contributions to our understanding of genetic drivers, disease initiation, and tumor progression (Huang et al, 2017; Li et al, 2025; McCreery et al, 2015; Nassar et al, 2015; Quintanilla et al, 1986; Wong et al, 2013), their long development timeline (6 or more months) make them less than ideal for therapeutic studies. Additionally, variability in the timing of tumor onset, heterogeneity in genetic drivers and immune responses between individual tumors, and the multiplicity of tumors in each animal (with single animals sometimes harboring upwards of 30 lesions) make it difficult to enroll animals in therapeutic studies. Because of these limitations in autochthonous models, syngeneic tumor models, in which cancer cell lines are transplanted into immune-competent syngeneic hosts, play a particularly important role in preclinical research across many cancer types, with the reliability of their growth kinetics making them a workhorse for testing the efficacy of new therapies or combinations.

However, while syngeneic murine models of melanoma are widely available and have contributed to substantial advancements in immunotherapy, relatively few syngeneic tumor models are available for cSCC. Many barriers to the efficacy of immunotherapy are cancer type-specific, and there is a need for mouse models that accurately recapitulate the unique features of each disease. Additionally, murine models are essential for studying immunotherapy. Because ICIs primarily act directly on T cells, they are effective only in immunocompetent hosts, making patient-derived xenografts—which grow only in mice with compromised immune systems—unsuitable models to study immunotherapy. As such, the lack of availability of syngeneic cSCC models represents a significant gap in the field.

Here, we describe the carcinogen-induced tumor (CIT) cell line panel, a series of murine cSCC cell lines, to address this need. The CIT lines are derived from DMBA/TPA-induced cutaneous SCCs, and they are syngeneic to the FVB strain. Notably, the CIT cell lines recapitulate many key features of the mutational profiles of human cSCC, including harboring cSCC-relevant driver mutations in *Hras* and *Trp53* and deletions of *Cdkn2a*. As a result of being chemically induced in immune-competent hosts, the CIT lines carry a moderate to high mutation burden comparable to that of human cSCC (Chang and Shain, 2021; Pickering et al, 2014). The tumors that arise from the CIT lines exhibit a range of reproducible patterns of immune infiltration—from high to low T cell infiltrate—along with a corresponding range of responses to immunotherapy. Additionally, as a consequence of their high mutation burden, the CIT lines harbor endogenous neoantigens, which are mutant proteins—resulting from genetic mutations—that can be recognized by T cells (Chen and Mellman, 2017). Neoantigens enable T cells to identify tumor cells as “foreign,” and this recognition is a critical central mechanism of the anti-tumor immune response that is enhanced by ICI therapy (Chen and Mellman, 2017; Gubin et al, 2014). The identification of these neoantigens—two of which we describe in this manuscript—provides an additional valuable tool to facilitate the study of tumor-specific T cells during immunotherapy responses in cSCC.

The CIT cell line series thus offers a panel of cSCC tumor models that exhibit a range of mutational burdens, immune infiltrates, and neoantigens, while being driven by cSCC-relevant genomic drivers and exhibiting histopathologic features characteristic of cSCC. These properties make the CIT lines a valuable set of preclinical models for investigating tumor biology and responses to therapy, particularly immunotherapy, in cSCC.

## Results

### CIT lines display a range of reproducible immune infiltration phenotypes

**Figure 1:**
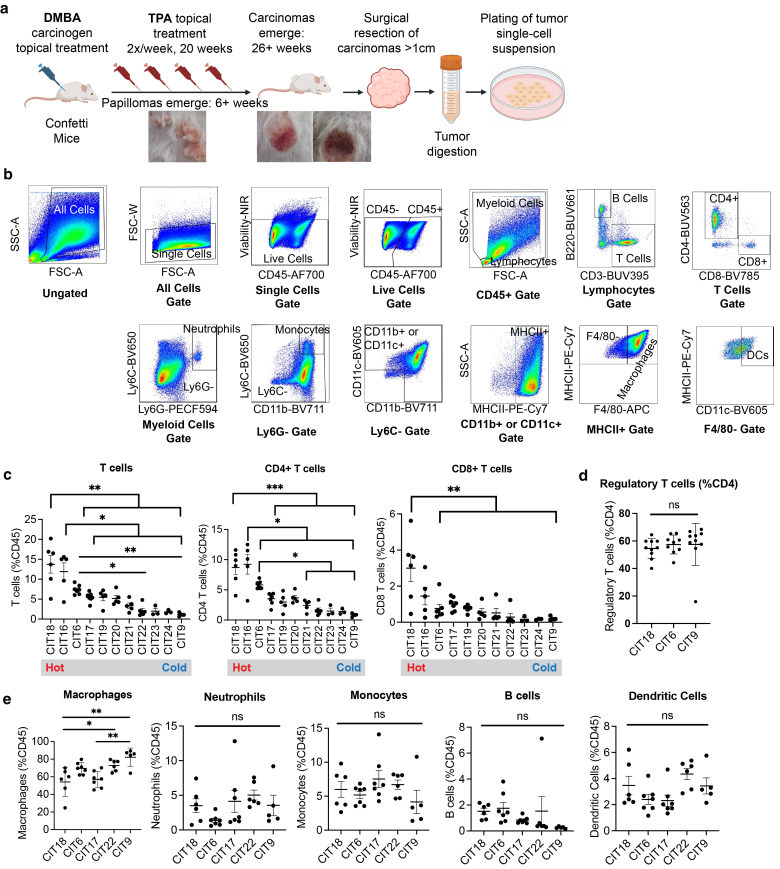
**Immune infiltration profiles of tumors arising from engraftment of CIT lines.** (**a**) Schematic of CIT cell line generation. Tumors were induced with the DMBA/TPA carcinogenesis model. Cell lines were established from malignant carcinomas by digesting resected tumors into a single cell suspension and subsequently growing cells in vitro under standard culture conditions. **(b)** Flow cytometric gating strategy of main immune cell types. Immune analysis of a CIT6 tumor is shown as representative data. **(c)** Quantification of total T cells, CD4+ T cells, and CD8+ T cells by flow cytometry in tumors from each CIT line (n = 5–7 mice per group; ∗ *P* < .05, ∗∗ *P* < .01, ∗∗∗ *P* < .001) **(d)** Abundance of Foxp3+CD25+ Tregs as percentage of CD4+ T cells in CIT18, CIT6 and CIT9 tumors. (n = 9–10 mice per group, ns) **(e)** Quantification of macrophages, neutrophils, monocytes, B cells, and dendritic cells by flow cytometry in tumors from 5 selected CIT lines. (n = 5–7 mice per group; ∗ *P* < .05, ∗∗ *P* < .01, ns) Statistical analysis in (c–e) was performed by one-way ANOVA with Tukey post-hoc test; single bars represent significance between two CIT lines; brackets represent significance between one CIT line and multiple others; single bars denoted “ns” represent lack of significance across the cohort. For example, the ∗∗ designation above the first bracket in Figure 1c denotes that the total number of T cells in CIT18 is significantly different from each CIT6, CIT17, CIT19, CIT20, CIT21, CIT22, CIT23, CIT24, and CIT9, with *P* < .01. Error bars in (c and e) represent mean and standard error. Error bars in (d) represent the mean and SD. CIT, carcinogen-induced tumor; DMBA, 7,12-dimethylbenzanthracene; ns, not significant; TPA, 12-O-tetradecanoylphorbol-13-acetate; Tregs, regulatory T cells.

The CIT cell lines were derived from skin carcinomas that arose by topically treating *K5-CreER*^*T2*^-*Confetti* FVB/N mice with a single dose of DMBA followed by TPA twice weekly for 20 weeks, using a well-established skin carcinogenesis protocol (McCreery et al, 2015; Wong et al, 2013) (Figure 1a). In the DMBA/TPA model, benign papillomas arise after about 6 weeks of TPA treatment, and approximately 10% of these progress to malignant carcinomas. Primary carcinomas were resected when they were approximately 1 cm in longest diameter, digested into a single-cell suspension, and grown under standard tissue culture conditions. Out of the primary carcinomas we attempted to culture, we had an approximately 65% success rate of achieving a stably growing cell line. Each cell line was established in tissue culture for > 10 passages. Implantation of 1.25 × 10^5^ tumor cells into the subcutaneous flank of FVB mice gave rise to tumors with 100% penetrance, and we subsequently profiled the histopathologic and immune characteristics of these tumors.

We carried out immune profiling of eleven CIT cell lines by flow cytometry, quantifying the number of total T cells, CD4+ and CD8+ T cells in tumors from each CIT model (Figure 1b and c). These 11 CIT cell lines came from a total of 10 mice, with CIT21 and CIT23 being derived from two distinct carcinomas from the same mouse, while all other CIT cell lines came from different mice. We found each CIT line gave rise to tumors with a consistent pattern of immune infiltration, spanning the range of immune “hot” (highly infiltrated by T cells relative to all CD45+ immune cells, eg, CIT18) to immune “cold” (poorly infiltrated by T cells, eg, CIT9) (Figure 1c). Interestingly, we observed a consistent bias in the T cell compartment toward CD4+ T cells, about half of which were Tregs (Figure 1d). The bias towards infiltration of CD4+ T cells over CD8+ T cells mirrors clinical observations of immune infiltration in human cSCC (Freeman et al, 2014; Kosmidis et al, 2010; Mühleisen et al, 2009). We selected 5 CIT lines, including representation of both hot and cold models (with high or low T cell infiltrate, respectively, based on Figure 1c), for more extensive immune profiling. On the whole, we found the myeloid compartment of CIT tumors was strongly biased toward macrophages (CD11b+MHCII+F4/80+), with relatively fewer Ly6C+ and Ly6G+ myeloid cells (monocytes and neutrophils, respectively) and dendritic cells (CD11c+MHCII+) (Figure 1b and e). Macrophage infiltration was highest in the two coldest (lowest T cell infiltrate) CIT models, CIT22 and CIT9, where they constituted the majority of tumor-infiltrating CD45+ immune cells. Conversely, hot models CIT18 and CIT6 exhibited a non-significant trend towards higher B cell infiltration (Figure 1e). There was no clear trend between hot and cold tumors for Ly6G^hi^Ly6C^mid^ neutrophils, Ly6C^hi^CD11b+ monocytes, or CD11c+MHCII+ dendritic cells (Figure 1e).

**Figure 2:**
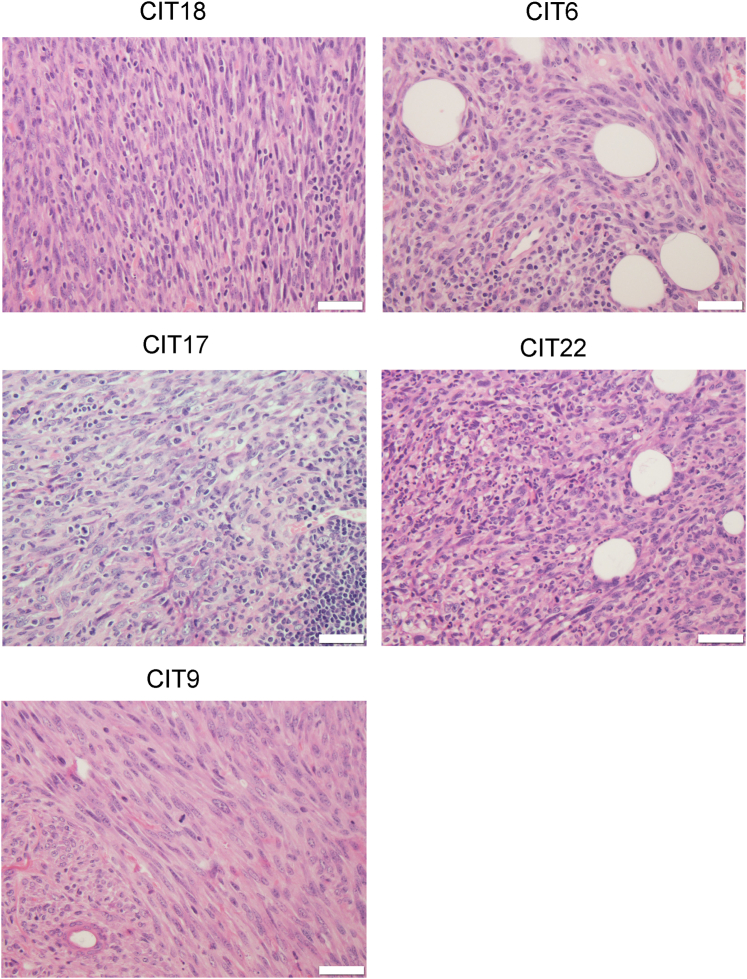
**Histology of tumors arising from engraftment of CIT lines.** Representative images of tumors from selected CIT lines stained with H&E for histological analysis. Scale bar = 20 mm. CIT, carcinogen-induced tumor.

**Figure 3:**
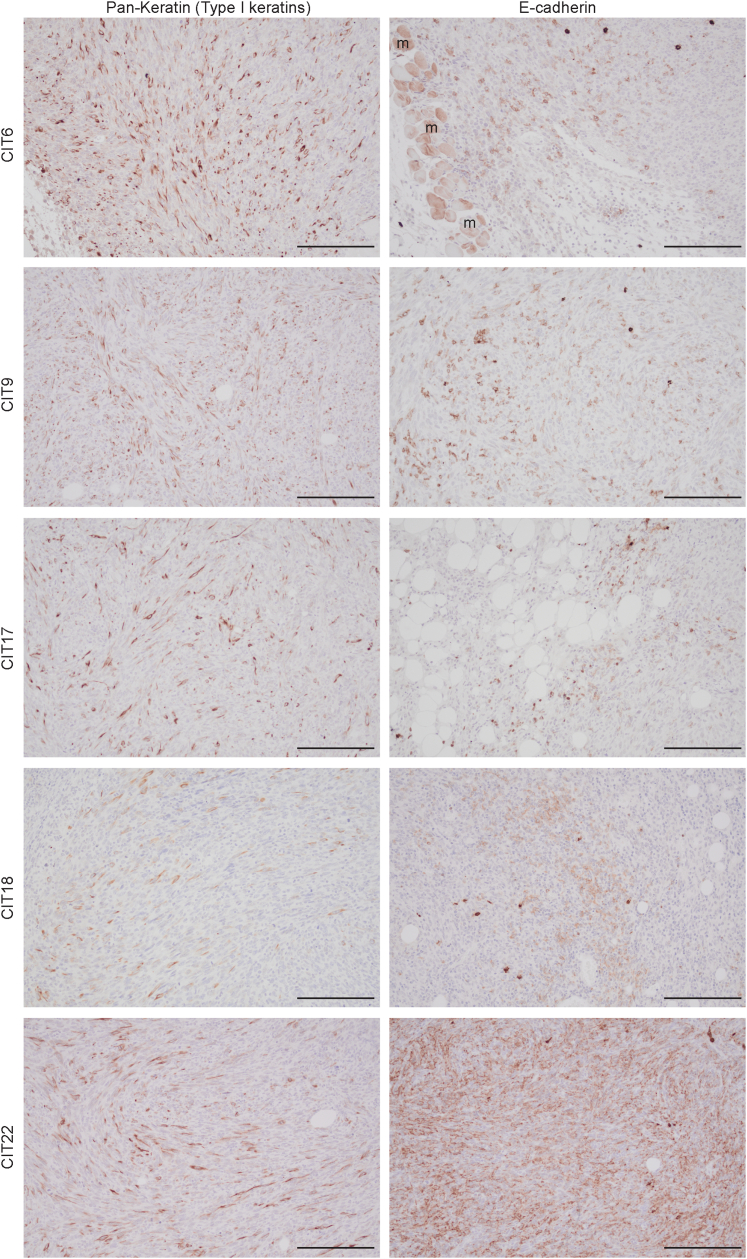
**Tumors arising from CIT cell line engraftment stain positive for keratins and E-cadherin.** Tumors arising from CIT6, CIT9, CIT17, CIT18, and CIT22 have patchy positive staining for anti-mouse keratin I cocktail (cytoplasmic pattern) and E-cadherin (plasma membrane pattern). All images were taken at 20 x magnification. Scale bar = 120 mm. CIT, carcinogen-induced tumor; m, skeletal muscle.

Two board-certified pathologists reviewed the tumor histology for the same five selected CIT tumor models. Tumors from these five CIT models were poorly differentiated, spindled, arranged in fascicles, and highly mitotic (Figure 2). Some CIT18 tumors contained pleomorphic, rhabdoid, or epithelioid malignant cell morphologies. Among the hot models, CIT18 and CIT17 tumors contained brisk tumor-infiltrating lymphocyte infiltration, while CIT6 tumors contained focal clusters of tumor-infiltrating lymphocytes or tumor-infiltrating lymphocytes localized to the edge of the tumor. In comparison, tumors arising from the cold lines CIT9 and CIT22 displayed very little tumor-infiltrating lymphocyte infiltration. CIT6, CIT17, CIT18 and CIT22 tumors contained diffuse infiltration of polymorphonuclear cells, while polymorphonuclear cells in cold CIT9 tumors were largely restricted to the edge of the tumors. Immunohistochemical staining with pan-keratin and E-cadherin showed that patchy and scattered cells stained positive for a pan-keratin cocktail (type I keratins) and for E-cadherin (Figure 3). The staining profile mirrors the frequency of positively staining cells in clinical spindle cell SCC. Altogether, we find that CIT18, CIT6, CIT17, CIT22, and CIT9 cell lines give rise to tumors that recapitulate the histologic features of high-grade, poorly differentiated human cSCC (Cocuz et al, 2024; Read et al, 2024; Smoller, 2006), and each exhibits characteristic patterns of immune infiltration congruent with flow cytometry–based immune profiling.

### Genomic profiles of CIT lines recapitulate human SCC

**Figure 4:**
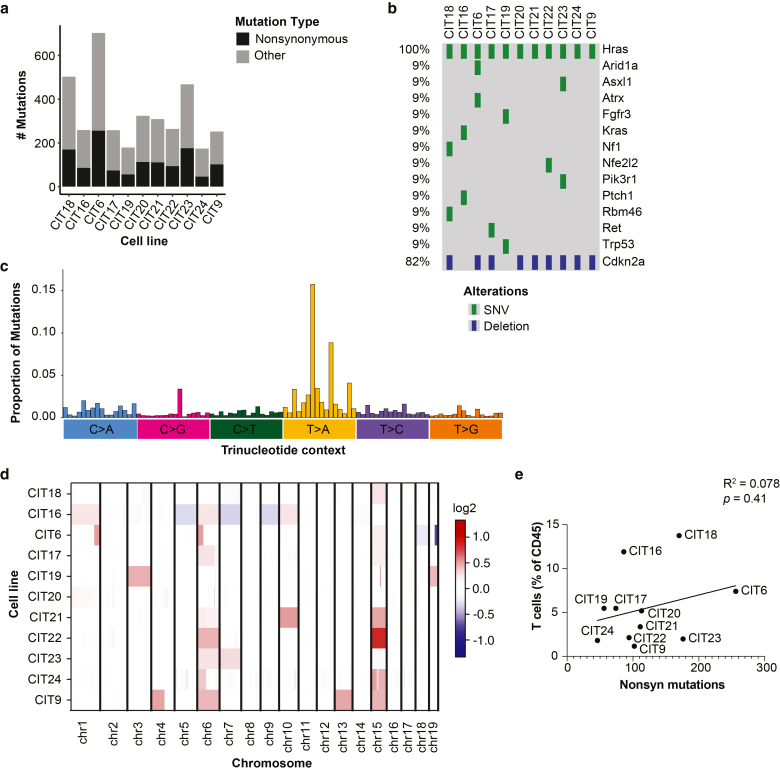
**Genetic profiling of CIT lines.** (**a**) Number of total and non-synonymous mutations per cell line, based on exome sequencing. **(b)** Genes mutated in CIT lines, which are known to be commonly mutated in human SCC, and CIT lines with *Cdkn2a* deletions. **(c)** Frequencies of mutations observed in each of 96 possible trinucleotide contexts for all mutations in CIT cell lines. Trinucleotide contexts, arranged on the x-axis, are grouped by the base pair change of the mutation. Highest peaks are observed at CTG > A and GTG > A. **(d)** CNV alterations detected in each chromosome in CIT lines based on exome sequencing. Red denotes copy number gains; blue denotes copy number losses. **(e)** Scatter plot showing the relationship between non-synonymous mutations (based on exome sequencing) and T cell infiltrate (based on flow cytometry) for CIT lines. There is no significant correlation between these metrics. Statistical analysis in **(e)** was performed with simple linear regression. CIT, carcinogen-induced tumor; CNV**,** copy number variation; SCC, squamous cell carcinoma.

All eleven CIT cell lines were subjected to exome sequencing, to a median depth of 153X. Consistent with previous sequencing of DMBA/TPA tumors by ourselves and others (Li et al, 2025; McCreery et al, 2015; Nassar et al, 2015), CIT cell lines were genomically characterized by *Hras* driver mutations, *Cdkn2a* losses, a substantial mutation burden, and whole-chromosome copy number alterations. The mutation burden among the CIT lines ranged from 174 to 703 mutations (5.2–21.0 mutations/Mb; average of 10 mutations/Mb), including 46 to 256 non-synonymous mutations (Figure 4a, Table 1, Supplementary Table S1). Ten of our CIT lines exhibited a *Hras* Q61L mutation, which was one of the only mutations shared between multiple cell lines. One cell line, CIT16, did not exhibit a *Hras* Q61L mutation but instead exhibited a *Hras* G12V as well as a *Kras* G13R mutation (Figure 4b, Supplementary Table S1). Seven CIT lines displayed additional non-synonymous mutations in driver genes known to be mutated in human cSCC, including *Trp53* and *Fgfr3*, while nine CIT lines harbored a deletion at the *Cdkn2a* locus (Figure 4b, Supplementary Table S1). The majority of total mutations were T > A transversions, with a particular prevalence of CTN > A mutations (with N representing any nucleotide) consistent with the known mutagenic signature of DMBA (Li et al, 2025; McCreery et al, 2015; Nassar et al, 2015) **(**Figure 4c**)**. While this signature is not identical to the C > T mutations induced by UV and typically found in cSCC (Chang and Shain, 2021; Pickering et al, 2014), we note that the genes mutated in the CIT lines were nonetheless cSCC-relevant driver genes, including mutations at classical *Hras* hotspots in codons 12 and 61. The CIT lines also harbored whole-chromosome alterations, primarily amplifications, especially on chromosomes 6 and 15 **(**Figure 4d**)**. Chromosome 6 amplifications are common in DMBA/TPA-induced skin carcinomas (McCreery et al, 2015). Chromosome 15 amplifications were also previously observed, but they were more heavily represented in the CIT lines than in a previously sequenced cohort of primary tumors (McCreery et al, 2015), suggesting perhaps lines with this amplification were particularly compatible with establishment in tissue culture. Interestingly, we observed only one whole-chromosome amplification each of chromosomes 1 and 7. We previously reported that chromosome 1 and 7 amplifications are common in DMBA/TPA carcinomas with an epithelial-like “squamous” morphology, but largely absent in DMBA/TPA carcinomas with a poorly differentiated “spindle” morphology (McCreery et al, 2015). The underrepresentation of these alterations is perhaps not surprising given that the CIT lines primarily give rise to poorly differentiated tumors, but it may reflect that well-differentiated tumors with squamous morphology were also underrepresented in the cell lines we were able to establish.

**Table 1:** Number of Total and Non-Synonymous Mutations Per CIT Cell Line

Cell Line	Donor Mouse Sex	Total Mutations (Nbr)	Total Mutations Per MB	Non-Synonymous Mutations (Nbr)
CIT18	Female	503	14.92	170
CIT16	Female	259	7.76	86
CIT6	Male	703	20.95	256
CIT17	Female	258	7.74	74
CIT19	Male	179	5.36	56
CIT20	Female	324	9.66	113
CIT21	Male	309	9.32	111
CIT22	Female	264	7.91	94
CIT23	Male	468	14.13	176
CIT24	Male	174	5.22	46
CIT9	Female	252	7.58	102

Abbreviations: CIT, carcinogen-induced tumor; MB, megabase; Nbr, number.

Number of total mutations, mutations per megabase, and non-synonymous mutations based on exome sequencing of CIT cell lines, as well as the sex of the donor mouse.

Given that mutation burden information and immune phenotypes were available for all eleven cell lines, we examined whether a relationship existed between these metrics. While two of the tumors with the highest T cell infiltration, CIT18 and CIT6, exhibited two of the three highest mutation burdens, we observed that the tumor model with the second-highest T cell infiltration, CIT16, had a relatively low mutation burden. CIT23 tumors, which had the second-highest mutation burden, were, by contrast, very poorly infiltrated by T cells. Thus, aligning with observations from patients, mutation burden alone was insufficient to predict the immune response to any given tumor, and there was no significant correlation between mutation burden and T cell infiltrate across CIT models (Figure 4e).

### Immunologically hot CIT lines respond to immunotherapy

**Figure 5:**
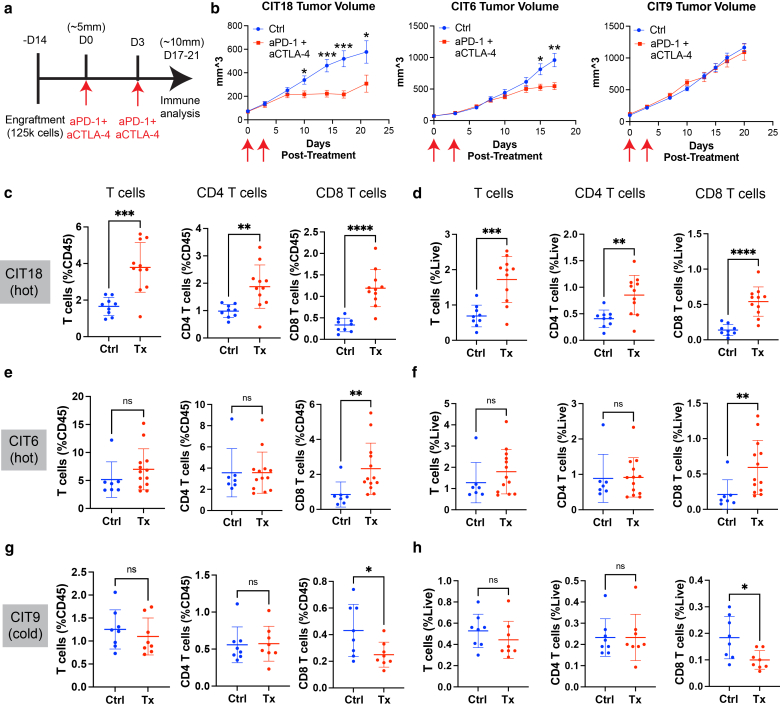
**Immunologically hot CIT lines are responsive to combined anti-PD-1 and anti-CTLA-4 ICI therapy.** (**a**) Schematic of the ICI treatment regimen on CIT tumor-bearing mice. **(b)** Average tumor volumes of immunologically hot CIT18 and CIT6 tumors and immunologically cold CIT9 tumors after combined ICI therapy, compared to tumors treated with isotype control antibodies. Error bars represent the mean and standard error. **(c and d)** Total T cell, CD4+ T cell, and CD8+ T cell infiltration of CIT18 tumors as a percentage of all CD45+ immune cells **(c)** and as a percentage of all live cells **(d)** for Ctrl or Tx tumors. **(e and f)** Total T cell, CD4+ T cell, and CD8+ T cell infiltration of CIT6 tumors as a percentage of all CD45+ immune cells (**e**) and as a percentage of all live cells (**f**) for Ctrl or Tx tumors. (**g and h**) Total T cell, CD4+ T cell, and CD8+ T cell infiltration of CIT9 tumors as a percentage of all CD45+ immune cells (**g**) and as a percentage of all live cells (**h**) for Ctrl or Tx tumors. All statistical analyses were performed with Welch’s *t-test*, n = 7**–**13 mice per group. ∗ *P* < .05, ∗∗ *P* < .01, ∗∗∗ *P* < .001, ∗∗∗∗ *P* < .0001. Error bars in (**c–h**) represent mean and SD. CIT, carcinogen-induced tumor; Ctrl, control-treated; ICI, Immune checkpoint inhibitor; Tx, ICI-treated. CIT, carcinogen-induced tumor; ICI, Immune checkpoint inhibitor.

Because our CIT lines exhibited moderate to high tumor mutation burdens, we hypothesized they would carry neoantigens and would be sensitive to ICIs. We tested the efficacy of a combination ICI regimen—anti-PD-1 plus anti-CTLA-4, administered as two doses three days apart—on CIT18, CIT6, and CIT9 tumors (Figure 5a). We selected these three tumor lines as representatives of the highest and lowest T cell infiltration patterns in our initial immune profiling (Figure 1c).

CIT18 and CIT6 tumors—both of which we had observed to be immunologically hot—grew significantly slower than controls when treated with anti-PD-1 plus anti-CTLA-4 (hereafter, “ICI”) **(**Figure 5b**)**. This effect was observed in both male and female mice. Conversely, immunologically cold CIT9 tumors displayed no difference in growth between ICI-treated and control tumors (Figure 5b), and this observation was also consistent between male and female mice. We carried out immune profiling of ICI- and control-treated tumors to interrogate how ICI remodeled the immune response in these models. CIT18 tumors had the strongest response to ICIs, both in terms of the reduction in growth and in the increase in tumor-infiltrating T cells. Total T cells, CD8+ T cells and CD4+ T cells were all more abundant in ICI-treated CIT18 tumors, both as a fraction of total CD45+ immune cells (Figure 5c) and of total live cells (Figure 5d). CIT6 tumors, which showed a more modest response to ICI than CIT18, only exhibited an influx of CD8+ T cells following ICI, while CIT9 tumors showed no increase in overall T cell infiltration. (Figure 5e–h).

**Figure 6:**
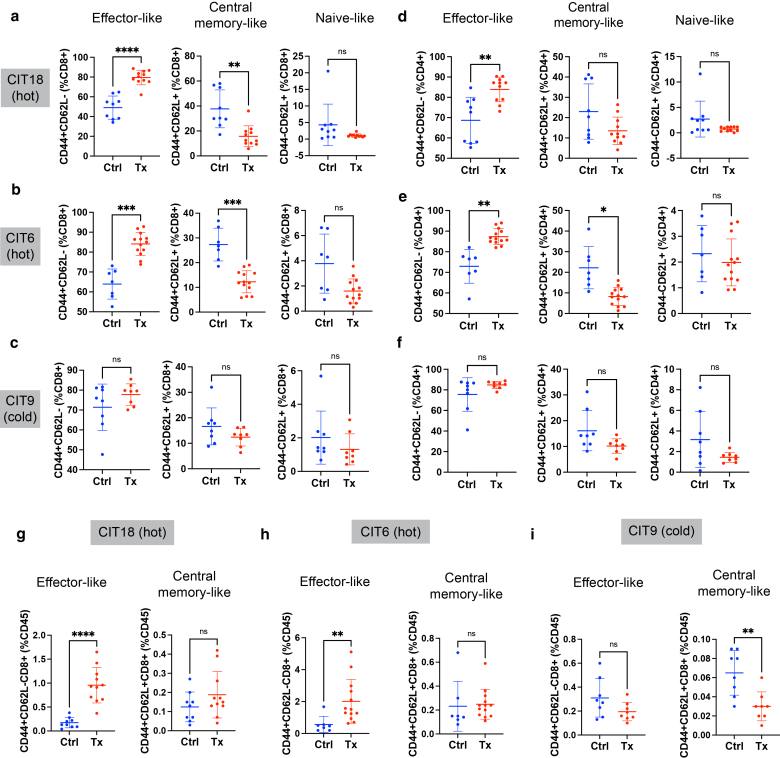
**Tumor-infiltrating T cell effector subsets after treatment with ICI.** (**a–c**) Abundance of effector subsets of CD8+ T cells defined by the combinatorial expressions of CD44 and CD62L in CIT18 (**a**), CIT6 (**b**), and CIT9 (**c**) tumors. **(d–f)** Abundance of effector subsets of CD4+ T cells defined by the combinatorial expressions of CD44 and CD62L in CIT18 (**d**), CIT6 (**e**), and CIT9 (**f**). **(g–i)** Infiltration of CD44+CD62L- “effector-like” CD8+ T cells and CD44+CD62L+ “central memory-like” CD8+ T cells as percentage of all CD45+ immune cells in CIT18 (**g**), CIT6 (**h**) and CIT9 (**i**) tumors. All statistical analyses were performed with Welch’s *t-test*, n = 7**–**13 mice per group. ∗ *P* < .05, ∗∗ *P* < .01, ∗∗∗ *P* < .001, ∗∗∗∗ *P* < .0001. Error bars represent the mean and SD. CIT, carcinogen-induced tumor; ICI, Immune checkpoint inhibitor.

**Figure 7:**
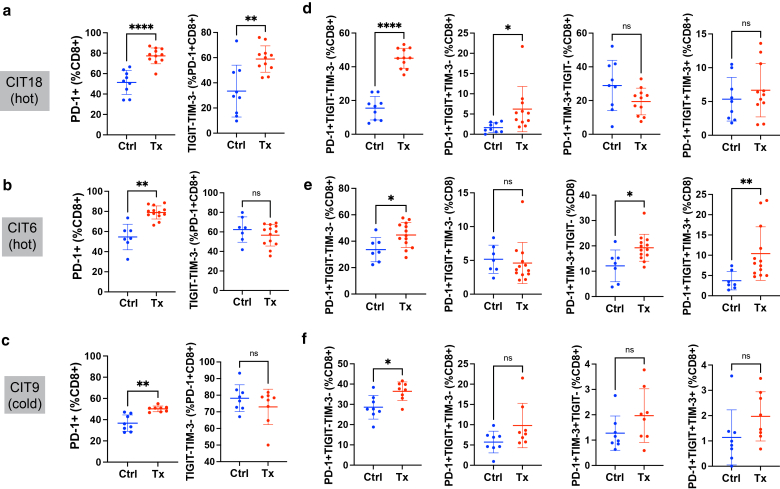
**Tumor-infiltrating T cell activation and exhaustion markers after treatment with ICI.** (**a–c**) Abundance of CD8+ T cells expressing PD-1, and frequency of “activated” PD-1+Tim3 TIGIT- CD8+ T cells in CIT18 (**a**), CIT6 (**b**) and CIT9 (**c**) tumors. **(d–f)** Abundance of CD8+ PD-1+ T cells expressing specific combinations of TIGIT and Tim3 exhaustion markers in CIT18 (**d**), CIT6 (**e**), and CIT9 (**f**) tumors. All statistical analyses were performed with Welch’s *t-test*, n = 7**–**13 mice per group. ∗ *P* < .05, ∗∗ *P* < .01, ∗∗∗ *P* < .001, ∗∗∗∗ *P* < .0001. Error bars represent the mean and SD. CIT, carcinogen-induced tumor; ICI, Immune checkpoint inhibitor.

ICI treatment also altered the activation profile of tumor-infiltrating T cells (Figure 6a–i). Effective ICI treatment in CIT18 and CIT6 tumors was accompanied by an increase in the percentage of T cells that exhibited a CD44+CD62L- “effector-like” phenotype, evident among both CD8+ T cells (Figure 6a–b) and CD4+ T cells (Figure 6d–e). Notably, in hot tumors, this increase in “effector-like” CD8+ T cells resulted in a skewing of the CD8+ compartment away from a CD44+CD62L+ “central memory-like” phenotype, even though the abundance of central memory-like CD8+ T cells as a percentage of all CD45+ immune cells was unchanged (Figure 6g and h). In contrast, ICI treatment in cold CIT9 tumors was unsuccessful at shifting the phenotype of either CD8+ or CD4+ T cells (Figure 6c, f, i). We further examined the expression of PD-1, which is present on both activated and exhausted T cells, and Tim3 and TIGIT, both of which mark exhaustion, on CD8+ T cells. The proportion of tumor-infiltrating CD8+ T cells expressing PD-1 increased after ICI treatment in all three tumor models (Figure 7a–f). Interestingly, the “activated” PD-1+Tim3-TIGIT- T cell population was the primary CD8+ population to increase in CIT18 tumors following ICI (Figure 7a and d), and this corresponded to the best tumor growth control. By contrast, in CIT6 tumors, which showed more modest tumor growth control, the proportion of “activated” PD-1+Tim3-TIGIT- CD8+ T cells did not increase as a fraction of total PD-1+ CD8+ T cells (Figure 7b). Instead, there was an increase in the proportion of PD-1+ CD8+ T cells with an exhausted (PD-1+Tim3+ or PD-1+Tim3+TIGIT+) phenotype after ICI treatment (Figure 7e). Together, these data show that CIT tumor models exhibit a range of responses to immunotherapy and that the degree of sensitivity appears to be approximately correlated with the level of T cell infiltration in untreated tumors.

### CIT tumors display identifiable and immunotherapy-sensitive neoantigens

**Figure 8:**
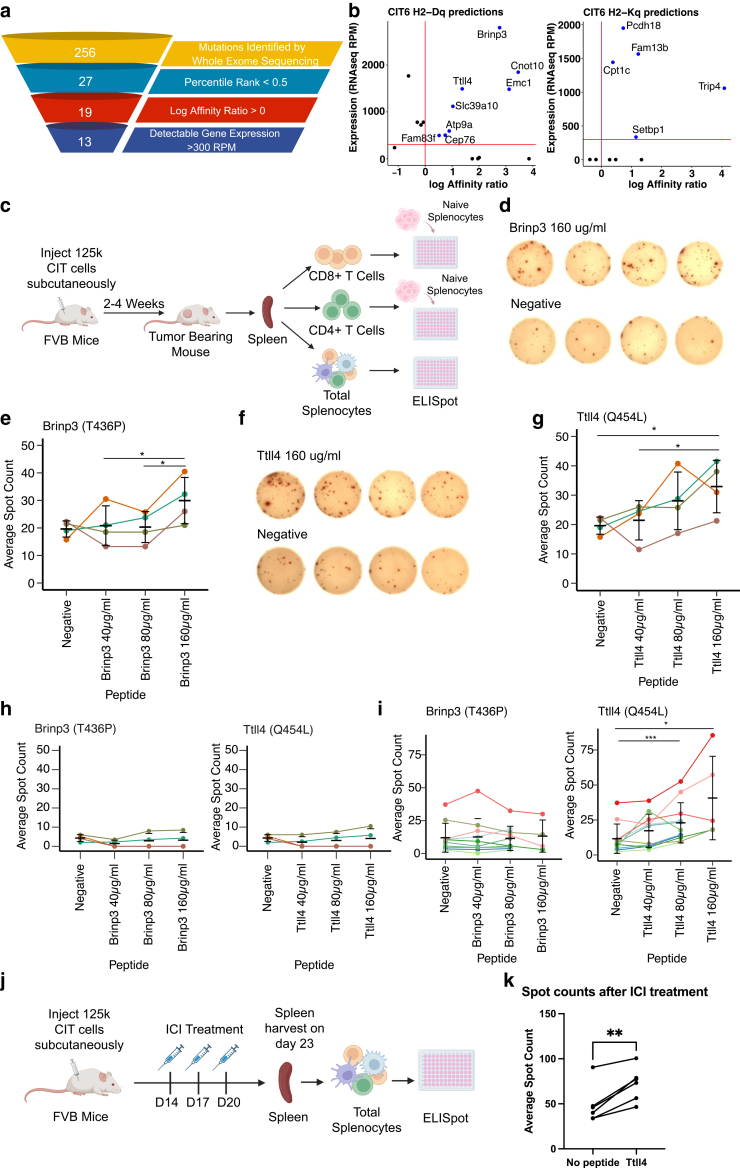
**CIT lines express identifiable neoantigens whose cognate CD8+ T cells are responsive to immunotherapy.** (**a**) Selection criteria for CIT6 mutations predicted to generate neoantigens. **(b)** Predicted peptide log affinity ratio (affinity ratio is defined as the ratio between predicted binding affinity of mutant versus wild-type sequence) and gene expression level of mutations predicted to be top CIT6 candidate neoantigens, shown for H2-Dq and H2-Kq alleles. Affinity predictions were generated using the NetH2Pan algorithm. **(c)** Schematic of IFNγ ELISpot assay. For screening of CD8+ T cell and CD4+ T cell responses, CD8+ or CD4+ T cells were isolated from the spleens of tumor-bearing mice and co-cultured with total splenocytes from a healthy tumor-naïve mouse. **(d)** Representative IFNγ ELISpot images of the assay performed with CD8+ T cells from the spleen of CIT6-bearing mice cultured with 160ug/ml of Brinp3 peptide CPAFLPCTV (top row) and negative control (no peptide, bottom row). **(e)** Quantification of average IFNγ ELISpot spot counts for CD8+ T cells from the spleen of CIT6-bearing mice cultured with different concentrations of Brinp3 peptide CPAFLPCTV. Each colored line represents data points from a different mouse (n = 4 mice). **(f)** Representative IFNγ ELISpot images of the assay performed with CD8+ T cells from the spleen of CIT6-bearing mice cultured with 160ug/ml of Ttll4 peptide VPPSSLLPL (top row) and negative control (no peptide, bottom row). **(g)** Quantification of average IFNγ ELISpot spot counts for CD8+ T cells from the spleen of CIT6-bearing mice cultured with different concentrations of Ttll4 peptide VPPSSLLPL. Each colored line represents data points from a different mouse (n = 4 mice). **(h)** Spot counts from ELISpot assays performed with CD4+ T cells isolated from the spleens of CIT6-bearing mice and cultured with splenocytes from tumor-naïve mice loaded with increasing concentrations of Brinp3 and Ttll4 peptides. Each colored line represents data points from a different mouse (n = 4 mice). **(i)** Spot counts from ELISpot assays performed with total splenocytes from CIT6-bearing mice cultured with increasing concentrations of Brinp3 and Ttll4 peptides. Each colored line represents data points from a different mouse (n = 10**–**11 mice). **(j)** Schematic of ICI treatment regimen and ELISpot analysis. **(k)** Average spot counts of ICI-treated splenocytes cultured with 80 ug/ml of Ttll4 peptide vs negative control. Each line represents data points from a different mouse (n = 6 mice). Statistical analysis in panels e, g, h, i, and k was done by a paired *t-*test. ∗ *P* < .05, ∗∗ *P* < .01. Error bars in panels e, g, h and i represent mean and SD. CIT, carcinogen-induced tumor; ICI, Immune checkpoint inhibitor.

Cytotoxic CD8+ T cells recognize tumor cells by using their TCR to detect neoantigens, which are fragments of mutant proteins that arise from genetic mutations in the tumor (Chen and Mellman, 2017; Gubin et al, 2014), and which allow T cells to see tumor cells as “foreign.” Given that improvement in the CD8+ T cell response corresponded to effective tumor control following ICI, we next sought to identify the neoantigens recognized by CD8+ T cells during this response. We focused here on the CIT6 tumor model, which exhibited the intermediate response to ICI among the three models tested and thus may be most representative of patient tumors that are recognized by the host immune response but benefit only marginally from current ICI strategies. We employed the NetH2pan algorithm (DeVette et al, 2018) to predict the binding strength between the H2-D^q^ and H2-K^q^ MHCI alleles carried by FVB/N mice and the neoepitopes predicted to be generated by all non-synonymous mutations identified in our exome sequencing analysis. We identified 27 top binders, and these were further filtered to prioritize those for which the mutant sequence was predicted to exhibit better binding than the wild-type sequence, and for which we could detect expression of the gene by RNAseq (Figure 8a and b, Table 2).

**Table 2:** List of Filtered Peptide Candidates for CIT6 Neoantigens

Gene	Amino Acid Change	Peptide	Allele	Percentile Rank
Slc39a10	S286I	LPEHIGHEL	Dq	0.0225
Ttll4	Q454L	VPPSSLLPL	Dq	0.1367
Brinp3	T436P	CPAFLPCTV	Dq	0.2119
Cep76	P158A	VPCACEADF	Dq	0.4240
Atp9a	I216F	EPNIDFHNF	Dq	0.4662
Fam83f	Q4L	VPALPTESL	Dq	0.1392
Emc1	H307L	SHYALLHYL	Dq	0.2106
Cnot10	A543P	VPLALGDNL	Dq	0.0063
Fam13b	D766H	QEKLARHL	Kq	0.1266
Setbp1	N1160Y	HDYLSGLF	Kq	0.2688
Pcdh18	M631I	NEENIFII	Kq	0.1341
Cpt1c	Q29H	HEIYLCAL	Kq	0.3203
Trip4	Q423L	HQHQLRIL	Kq	0.3234

Abbreviations: CIT, carcinogen-induced tumor.

List of all neoantigen peptide candidates that met the criteria specified in Figure 8a. Percentile ranked based on the NetH2pan algorithm.

Candidate neoantigens identified through this pipeline were subsequently screened by in vitro IFNγ ELISPOT assay (Alspach et al, 2019; Castle et al, 2012). Whole splenocytes, CD8+ T cells, or CD4+ T cells from the spleen of CIT6 tumor-bearing mice were incubated with synthetic peptides corresponding to each candidate epitope sequence (Figure 8c). We identified two peptides, CPAFLPCTV (resulting from a Brinp3 T436P mutation) and VPPSSLLPL (resulting from a Ttll4 Q454L mutation), which consistently elicited IFNγ production in sorted CD8+ T cells from CIT6-bearing mice in a peptide concentration-dependent manner (Figure 8d–g). These peptides elicited no response from sorted CD4+ T cells (Figure 8h). When the peptide assay was carried out with whole splenocytes (no prior sorting of CD8+ or CD4+ T cells), the dose-dependent response to VPPSSLLPL (Ttll4 Q454L mutation) was still observed, but the response to CPAFLPCTV (Brinp3 T436P mutation) was not detectable above background (Figure 8i).

We next interrogated the effect of ICI therapy on the T cell response to VPPSSLLPL, focusing on VPPSSLLPL because its response was more clearly detected in whole splenocytes **(**Figure 8i**)**. CIT6 tumors were treated with three doses of anti-PD-1 and anti-CTLA-4 combination therapy (Figure 8j), and an IFNγ ELISPOT assay was carried out on whole splenocytes harvested 3 days after the final dose of ICI. Splenocytes from ICI-treated mice produced a robust IFNγ response when incubated with VPPSSLLPL compared to no peptide (Figure 8k), indicating that the response to the neoantigen VPPSSLLPL is prominent during immunotherapy response.

## Discussion

We have established the CIT lines as a series of *Hras*-driven cSCC tumor lines, and here present histologic, genomic, and immune characterization of these cell lines. The CIT lines carry genomic drivers relevant to human cSCC, as well as a moderate-to-high mutation burden (Chang and Shain, 2021; Pickering et al, 2014). We show that each CIT line exhibits a characteristic immune infiltrate, with individual cell lines ranging from immune hot to immune cold. Immune hot CIT lines CIT6 and CIT18 exhibit partial responses to an immunotherapy regimen of anti-CTLA-4 and anti-PD-1. We have also identified two neoantigens that elicit CD8+ T cell responses in the CIT6 cell line. Because neoantigens are typically unique to each tumor—in both mouse models (Castle et al, 2012; Kreiter et al, 2015) and human patients (Ott et al, 2017; Sahin and Türeci, 2018; Sahin et al, 2017; Wolf and Sameuls, 2023)—and must be identified in order to enable tracking of tumor-specific T cells, our identification of these neoantigens provides a valuable tool that increases the utility of the CIT models for the field. Of note, while numerous neoantigens have been identified in tumor models syngeneic to C57BL/6 (Alspach et al, 2019; Castle et al, 2012; Matsushita et al, 2012; Roerden et al, 2024; Yadav et al, 2014), there has been a notable dearth of neoantigen identification in other mouse strains, including FVB, as well as a lack of neoantigens identified in cSCC models.

As noted above, the CIT cell lines are syngeneic to FVB mice. This stands in contrast to the majority of mouse models currently used for immunotherapy studies—across all cancer types—which are syngeneic to C57BL/6 mice. C57BL/6 mice are known to have a T helper type 1-biased immune response (Sellers et al, 2012). While this is likely beneficial for tumor control—and C57BL/6 mice are widely known for their resistance to tumorigenesis (Davie et al, 2007; Hennings et al, 1985)—it is not necessarily representative of the diversity of human immune responses. FVB mice, in contrast to C57BL/6, are known to exhibit T helper type 2-biased immune responses (Kim et al, 2012; Sellers et al, 2012) and to be readily susceptible to tumorigenesis. While no single mouse model or mouse strain can represent the full spectrum of human immunity, increasing the diversity of mouse strains used to model anti-tumor immune responses is essential to capture human diversity and understand the full spectrum of barriers to immunotherapy success that are found in patients. Finally, an additional feature of the CIT lines is that they were derived from tumors in *K5CreER*^*T2*^*-Confetti* mice (Reeves et al, 2018; Tanaka et al, 2025), and as such, cells can be labeled with fluorescent tags following exposure to Cre recombinase. The CIT lines thus provide a valuable series of models for immunotherapy research in cSCC.

We acknowledge several limitations of the CIT cell line panel. While the CIT lines are driven by mutations in *Hras* (as well as in *Kras*, in the case of CIT16), *RAS*-driven tumors represent only a subset of human cSCC, comprising 10–20% of cSCCs (Chang and Shain, 2021; Pickering et al, 2014). There remains a need for additional models recapitulating the full spectrum of cSCC drivers, including *Notch* and *Fat1* alterations, as well as for additional models harboring *Trp53* mutations, as only CIT19 in our panel carries an alteration in this common cSCC driver. Additionally, human cSCC typically carries a DNA damage signature associated with UVB exposure, which is characterized by C > T and CC > TT mutations (Chang and Shain, 2021; Pickering et al, 2014) and is distinct from the predominantly T > A mutation signature of DMBA. This results in differences in specific point mutations, for example, *HRAS* mutations in human cSCC are found in codons 12, 13, 27, and 61 (Boukamp, 2005; Chang and Shain, 2021; Pickering et al, 2014; Popp et al, 2002), while the majority of CIT lines specifically carry an *Hras* Q61L mutation. We also observe from histologic review of CIT tumors that they are predominantly poorly differentiated and do not represent well-differentiated cSCC. In summary, while the CIT lines represent a valuable set of models for studying cSCC, we acknowledge that they only partially recapitulate the features of human cSCC, and future work may be needed to fill some of these remaining gaps and add to the breadth of models available.

Improving immunotherapy outcomes in advanced cSCC remains an unmet clinical need. The CIT panel of cutaneous SCC tumor lines provides an important set of tools to better understand the mechanisms behind immunotherapy resistance in cSCC, and to preclinically model novel approaches to overcoming ICI resistance and improving patients with cSCC outcomes.

## Materials and Methods

### Animals

*K5CreER-Confetti FVB/N* mice were maintained in an in-house colony. Eight-week-old wild-type FVB/N mice were purchased from Jackson Laboratories. All experiments were performed on 8–16-week-old mice. Any diversity in mouse age or sex was addressed by distributing age and sex cohorts evenly between different experimental arms.

### Carcinogenesis and cell line generation

To induce tumors, *K5CreER-Confetti FVB/N* mice were treated with 25 μg DMBA followed by TPA (200 μl of a 10^–4^ M solution in acetone) two times a week for 20 weeks. The CIT16 line only was induced by DMBA/TPA treatment of a *Confetti-LSLHrasG12V* mouse rather than a *K5CreER-Confetti FVB/N* mouse. To generate cell lines, carcinomas were resected at a size of > 1 cm in longest diameter, finely chopped, digested in DMEM (high glucose, pyruvate; Gibco Catalog# 11995) containing Collagenase I and IV and DNase I for 45 minutes at 37 °C, washed with PBS, plated in DMEM (Gibco Catalog# 11995) plus 10% heat-inactivated fetal bovine serum (FBS) and L-glutamine, with penicillin, streptomycin, and amphotericin B, and passaged until cells stably grew in culture. The success rate of achieving a stably growing cell line from a carcinoma was ∼ 65%.

### Cell line culture

Cells were cultured in D10 media, composed of 88% DMEM (Gibco Catalog# 11995), 10% heat-inactivated FBS (Gibco Catalog# A56698-11), 1% 1x Penicillin-Streptomycin (Gibco Catalog# 15140-122), and 1% 1x L-Glutamine 200 mM (Gibco Catalog# 25030-081). Heat-inactivated FBS was re-filtered before adding to the media.

Frozen vials of CIT lines were thawed by immersing in a 37 °C water bath, transferred aseptically to a biological safety cabinet, and pipetted dropwise into a 15 ml conical containing 1 ml of warmed D10 media, with an additional 3 ml of warmed D10 media added dropwise afterward. The cells were centrifuged at 500 g for 5 minutes, the supernatant was aspirated, the cells were resuspended in 1 ml of D10 media, and the cells were counted on a cell counter (Invitrogen Countess II). Within the biological safety cabinet, ∼1 × 10^6^ cells were plated dropwise onto a 10 cm treated tissue culture dish (Avantor Catalog# 734-2321) containing 10 ml of warmed D10 media. Cells were then transferred to a 37 °C incubator.

Initial passaging of plated cells occurred 48 hours after thawing, or when cells achieved 80-90% confluence. In biological safety cabinet, D10 media was aspirated, and cells were washed with ∼ 4 ml of PBS. PBS was aspirated, and cells were de-adhered by adding 4 ml of 0.25% Trypsin-EDTA (1X) (Gibco Catalog# 25200-056) and incubated at 37 °C for 5 minutes. Trypsinization was quenched by adding an additional 4 ml of D10 media. Cell suspension was centrifuged in a 15 ml conical tube at 500 g for 5 minutes, supernatant was aspirated, the cell pellet was resuspended in D10, and a portion of the resuspension was plated dropwise into a new tissue culture plate. For the initial passage after thawing, cells were typically replated at a 1:5 dilution, and for subsequent passages, cells were typically replated at a 1:8 dilution due to increased growth rate. These dilution values were adjusted as necessary because different CIT lines grew at different rates. Cells were passaged every 48 hours for a total of 4 to 5 passages (∼ 1.5 weeks after thawing) before being utilized for experiments or refrozen. Passaging numbers were recorded for each set of thawed cells.

To freeze cell aliquots, cells were trypsinized and centrifuged as described above and resuspended in 3 to 4 ml freezing solution composed of 90% FBS and 10% dimethyl sulfoxide (MP Biomedicals Catalog# 196055). Cell suspension was pipetted into 1.2 ml Cryogenic Vials (Corning Catalog# 430487); each 80–90% confluent 10 cm tissue culture plate yielded sufficient cells for 3 or 4 cryovials. Cryovials were transferred into a Corning CoolCell LX cell freezing container (Corning Catalog# 432138) and transferred to a –80 °C freezer for 48 hours before final transfer into a liquid nitrogen tank.

### In vivo experiments and immune checkpoint inhibitor treatment

A total of 1.25 × 10^5^ cells were suspended in PBS without Matrigel and injected subcutaneously into the dorsal flank of 8 to 16-week-old male and female FVB/N mice. Where appropriate, male and female mice were distributed evenly between experimental arms, although similar tumor growth rates were generally observed between sexes. For baseline flow cytometric immune profiling and histological analysis (Figures 1 and 2), tumors were harvested approximately 21 days post-engraftment, when tumors measured ∼ 1 cm in diameter. For ICI experiments (Figure 5, Figure 6, Figure 7), mice were enrolled in treatment 14 days post-tumor engraftment, when tumors grew to 25–100 mm^3^ in volume. Any mice with tumors < 25 mm^3^ or > 100 mm^3^ on the day(s) of enrollment were excluded from the experiment. Mice were treated with 200 μg anti-PD-1 (BioXCell Catalog# BE0146) and 200 μg anti-CTLA-4 (BioXCell Catalog# BE0131) or 200 μg isotype control (BioXCell Catalog# BE0089). Mice received two doses 3 days apart. Mice were harvested when tumors were ∼ 10 mm in length, typically 17–21 days post-ICI enrollment and 31–35 days post-tumor engraftment, or when an ethical endpoint was reached.

### Flow cytometric immune profiling

Harvested tumors were finely chopped, digested in DMEM (high glucose, pyruvate; Gibco Catalog# 11995) containing Collagenase IV (Sigma-Aldrich Catalog# 9001-12-1) and DNase I (Sigma-Aldrich Catalog# D5025-15KU) for 45 minutes at 37 °C, and filtered through 70 um filters. Cells were counted using a Countess III, and 5 million cells were plated and stained with antibodies and Live/Dead NIR stain. Antibodies used for flow cytometric analyses are listed in Table 3. Flow cytometry analysis was performed on a Cytek Aurora.

**Table 3:** List of Antibodies Used for Flow Cytometry Analysis

Target	Fluorophore	Clone	Dilution	Manufacturer	Catalog #
B220	BUV661	RA3-6B2	1:200	BD Biosciences	612972
CD11b	BV711	M1/70	1:100	BioLegend	101242
CD11c	BV605	N418	1:100	BioLegend	117334
CD16/CD32 (FcR Block)	N/A	2.4G2	1:167	Tonbo	70-0161-U500
CD3e	BUV395	145-2C11	1:62.5	BD Biosciences	563565
CD4	BUV563	GK1.5	1:200	BD Biosciences	612923
CD44	BV785	1M7	1:200	BioLegend	103059
CD45	AlexaFluor700	30-F11	1:400	BioLegend	103128
CD62L	FITC	MEL-14	1:200	Invitrogen	11-0621-81
CD8a	BV570	53-6.7	1:200	BioLegend	100739
CD8a	BV785	53-6.7	1:200	BioLegend	100750
F4/80	APC	BM8	1:100	BioLegend	123116
FoxP3	PE-Cy7	FJK-16s	1:50	eBioscience	25-5773-82
Live/Dead	NIR	N/A	1:1000	Invitrogen	L34976A
Ly6C	BV650	HK1.4	1:200	BioLegend	128049
Ly6G	PE-CF594	1A8	1:200	BD Biosciences	562700
MHCII	PE-Cy7	M5/114.15.2	1:400	BioLegend	107630
PD-1	BV605	29F.1A12	1:100	BD Biosciences	135220
TIGIT	BV421	1G9	1:100	BioLegend	142111
Tim3	APC	RMT3-23	1:100	BioLegend	119705

All antibodies, clone numbers, and conjugated fluorophores for antibodies used for flow cytometry.

### H&E histology

Harvested tumors were fixed in formalin for 24 hours, placed in cassettes (VWR Catalog# 18000), submerged in 70% ethanol, and submitted to ARUP Laboratories Research Histology Core Laboratory, where they were embedded in paraffin, cut into 4 μm-thick slices, stained with H&E, and mounted on slides. The 4 μm slides were then analyzed by a board-certified pathologist, AG.

### Immunohistochemistry

For pan-keratin and E-cadherin immunohistochemistry stains, additional unstained slides were cut by ARUP Laboratories from the same paraffin blocks used for H&E histology. The slides were submerged in CitriSolv clearing agent (Decon Laboratories Catalog# 5989-27-5), followed by baths of 100%, 95%, 80% and 70% ethanol. They were then treated with an antigen blocking solution composed of Tris Base (Goldbio Catalog# T-400-500), EDTA Disodium, dihydrate (Goldbio Catalog# E-210-100), TWEEN 20 (Sigma-Aldrich Catalog# 9005-64-5), and distilled water. Next, they were treated with peroxidase blocking solution composed of hydrogen peroxide solution (Sigma-Aldrich Catalog# 7722-84-1) and distilled water, and blocked with bovine serum albumin (Sigma-Aldrich Catalog# 9048-46-8). Slides were stained with the following primary antibodies: pan-keratin (Type I) (E6S1S) rabbit monoclonal antibody (Cell Signaling Technology Catalog# 83957); E-Cadherin (24E10) rabbit monoclonal antibody (Cell Signaling Technology Catalog# 3195). Slides were subsequently stained with ImmPRESS HRP Horse antirabbit IgG polymer detection kit, peroxidase (Vector Laboratories Catalog# MP-7401-15) and ImmPACT DAB EqV substrate, peroxidase (HRP) (Vector Laboratories Catalog# SK-4103-100), counter-stained with Mayer’s Hematoxylin Solution (Sigma-Aldrich Catalog# MHS32-1L) and washed in 100% ethanol. Slides were reviewed by board-certified pathologist ES. Images were taken on an Olympus BX41 equipped with an Olympus UPlanFL N 20x objective and an Olympus DP23 camera.

### Cell line sequencing and analysis

DNA was extracted from cultured cells from each cell line and from tails of the mice in which each tumor originated using the Qiagen DNeasy Blood & Tissue DNA purification kit (Qiagen Catalog# 69504). Whole exome sequencing was performed using the Agilent Sure Select Mouse Exon capture kit and sequenced to an average depth of 153X on an Illumina HiSeq X by MedGenome, Inc. Reads were trimmed with Cutadapt (v3.5) (Martin, 2011) and aligned to the mm10 mouse genome using BWA (v0.7.17) (Li and Durbin, 2009). Reads were deduplicated using MarkDuplicates and recalibrated using BaseRecalibrator from GATK (v4.1) (Van der Auwera and O’Connor, 2020). Mutations were called using MuTect2 (GATK v4.1) (Cibulskis et al, 2013; Van der Auwera and O’Connor, 2020) and Strelka2 (version 2.9.10) (Kim et al, 2018), using tails from the mice in which each tumor developed as the matched normal. Mutation calls were filtered using the following criteria: minimum read depth of 10 at the mutation position in both tumor and normal samples; minimum of 4 reads supporting the mutation call; for Strelka only, minimum quality QSS score of 25. Mutations that passed all filters with both callers were kept and were additionally filtered to remove any germline SNP detected in the panel of normals. Final mutation calls were annotated with Annovar (Wang et al, 2010). Copy number calling was performed with CNVkit (version 0.9.10) (Talevich et al, 2016), using all tails as a pooled normal. *Cdkn2a* deletions were confirmed by samtools (version 1.16) (Li, 2011) mpileups of all reads aligning to the *Cdkn2a* locus (chr4:89,274,541-89,294,653).

### Authentication of CIT lines

CIT lines can be authenticated using Sanger sequencing of non-driver, cell line-specific mutations with high variant allele frequency identified in each CIT line by exome sequencing (Supplementary Table S1). This authentication strategy has been implemented and validated for CIT6 and CIT18. DNA was extracted from excised CIT6 and CIT18 tumors using Qiagen DNeasy Blood & Tissue DNA purification kit (Qiagen Catalog# 69504). PCR was performed using custom primers to amplify mutations unique to each cell line. For CIT6 tumors: *Ttll4* A1361T (nonsynonymous mutation Q454L) mutations were amplified using forward primer CCGGATTCCCAGAGTGCTAC and reverse primer AATAAACCAAGGGCAGGGGG; and *Pik3r3* A65T (nonsynonymous mutation Y22F) mutations were amplified using forward primer CGAGCTTCCCTGTAACCCTC and reverse primer GCAATCTACCCCTCATCCCG. For CIT18 tumors: *Agl* A2498T (nonsynonymous mutation Q833L) mutations were amplified using forward primer TTCCGAAGAATCCCCACAGC and reverse primer TGCCGAACATGACAGTGGAA; *Kcnk3* T559A (nonsynonymous mutation F187I) mutations were amplified using forward primer CCAAAACCTGCAAAGTGGGG and reverse primer GTCATGAATCGCAGCACCAC; and *Slc44a5* T129A (synonymous mutation I43I) mutations were amplified using forward primer CCTGTCCTGCCTTTCGGAAT and reverse primer ATTCATAACCGCTGCCCCTT. After amplification, PCR products were validated by gel electrophoresis and subjected to Sanger sequencing. Sanger sequencing results were analyzed using ApE software developed by the Erik Jorgensen Lab of the University of Utah Department of Biology to confirm the presence of all expected mutations in each cell line.

### Neoantigen peptide prediction

Mutations identified by exome sequencing were used to construct wild-type and mutant amino acid sequences of all 7mer, 8mer, and 9mer peptides that included each identified single-nucleotide variant. The NetH2pan algorithm was used to predict binding affinity and percentile rank for each wild-type and mutant peptide sequence. This data was filtered using the following criteria: mutant peptide with a percentile rank < 0.5, log affinity ratio > 0 (affinity ratio defined as the ratio of predicted binding affinity of mutant sequence divided by wild-type sequence), and a gene expression > 300 RPM based on RNAseq of whole CIT6 and CIT9 tumors.

### ELISpot assays

Whole spleens were harvested from FVB mice and processed by crushing through a 70 μm mesh filter, pre-wet with 1X PBS, using a syringe plunger, and washed. Red blood cells were lysed for 5 minutes using ACK lysis buffer, and splenocytes were counted. Spleens from CIT6-bearing FVB mice were harvested 2 to 4 weeks after tumor inoculation. CD8+ T cells were isolated from whole spleens using EasySep^TM^ Mouse CD8+ T Cell Isolation Kits (STEMCELL Technologies Catalog# 19853), and CD4+ T cells were isolated from whole spleens using EasySep^TM^ Mouse CD4+ T Cell Isolation (STEMCELL Technologies Catalog# 19852).

IFNγ ELISpots were performed over a three-day assay. Multiscreen®HTS 96-well filtration plate(s) were coated with IFNγ capture antibody (clone AN-18, Catalog# 517902), diluted 1:500 in 1X PBS. Plates were wrapped in aluminum foil to equally distribute temperature and incubated overnight, or at least 18 hours, at 4 °C. The following day plates were washed twice with 200 μl RPMI-2 (RPMI with 2% FBS, 1% L-glutamate, 1% streptococcus and penicillin antibiotic solution, and 0.09% 2-Mercaptoethanol) and blocked for two hours with 200 μl of RPMI-20 (RPMI with 20% FBS, 1% L-glutamate, 1% streptococcus and penicillin antibiotic solution, and 0.09% 2-Mercaptoethanol) before splenocytes or T cells were added. For assays where total splenocytes were used, 500,000 splenocytes were plated per well. For assays using CD8+ and CD4+ T cells, 20,000 cells per well were co-cultured with 500,000 splenocytes from a non-tumor-bearing mouse to provide a source of antigen-presenting cells. Stimulation medium was prepared by diluting the synthetic peptide in RPMI-10 to the desired peptide concentration(s). All synthetic peptides used for these assays were ordered through GenScript. Peptides were reconstituted in DMSO or MilliQ water according to solubility testing provided by the manufacturer. Reconstituted peptides were aliquoted and frozen at –80 °C. The negative control consisted of MilliQ water and RPMI-10. Positive control contained 0.04% Phorbol 12-myristate 13-acetate and 0.09% Ionomycin in RPMI-10.

Plates were wrapped in aluminum foil and incubated undisturbed at 37 °C and 5% CO2 for 44 to 48 hours before developing using biotinylated anti-IFNγ detection antibody (clone R4-6A2, Catalog# 505704), streptavidin-HRP enzyme conjugation (Thermofisher, Catalog# 405210), and AEC (3-amino-9-ethyl-carbazole) substrate. AEC substrate was prepared by dissolving 20 mg of AEC in 2 ml of N, N-dimethylformamide (Sigma-Aldrich, Catalog# 227056), diluting in 0.1 M Acetate Solution, adding hydrogen peroxide, and filtering with a 0.45 μM filter. Spots were imaged using the Agilent BioTek Cytation7 and analyzed using the Gen5 software.

### Statistical analysis

To determine significant statistical differences, Welch’s *t-test* was used when comparing 2 unpaired groups, and one-way ANOVA with Tukey post-hoc test was used when comparing 3 or more unpaired groups. Paired analyses were performed by a paired *t-*test. Statistical analysis in Figure 4e was performed with simple linear regression. Hypothesis testing was performed using R open-source software (version 4.4.2) and Prism software (GraphPad version 10.4.2). Significant *P* values are marked as ∗ *P* < .05, ∗∗ *P* < .01, ∗∗∗ *P* < .001, ∗∗∗∗ *P* < .0001. Studies were not blinded.

Power calculations to determine appropriate cohort size were performed using G∗Power software (version 3.1.9.7) available online from UCLA Advanced Research Computing. For all experiments, Type I error probability α was set at 0.05, and group sizes were calculated to achieve 80% power. For experiments comparing 2 groups, Cohen’s effect size d was set at 1.5, and for experiments comparing 3 or more groups, Cohen’s effect size f was set at 0.6. For unpaired multi-arm experiments, including immune phenotyping in Figure 1, we required n = 5 mice per arm. For unpaired 2-arm experiments, including comparison of tumor size between ICI-treated and control mice in Figure 5, Figure 6, Figure 7, we required a minimum of n = 9 mice per arm. For ICI experiments, additional mice were injected to account for growth variability and to ensure a sufficient number of mice available for simultaneous enrollment into treatment arms, and additional mice were enrolled when available.

### Materials availability

All CIT cell lines will be made available to the community as per standard practice (for example, under a material transfer agreement or licensing agreement), and requests can be initiated by contacting the corresponding author or the University of Utah technology transfer office.

## Ethics Statement

All animal experiments were approved by the University of Utah Office of Comparative Medicine (IACUC #22-02003 and #00001945) or by the University of California San Francisco Laboratory Animal Resource Center (IACUC #AN187679). This work complies with all the relevant ethical regulations regarding animal research.

## Data Availability Statement

Exome sequencing of CIT lines is available in SRA (accession number PRJNA1129114). https://www.ncbi.nlm.nih.gov/bioproject/PRJNA1129114

## ORCIDs

Robert Letchworth: http://orcid.org/0009-0001-8943-2218

Miho Tanaka: http://orcid.org/0000-0002-0905-9346

Alina A Barnes: http://orcid.org/0009-0000-7469-5560

Lotus Lum: http://orcid.org/0000-0003-4751-0764

Savannah Hughes: http://orcid.org/0009-0007-3593-6756

Grant Schlauderaff: http://orcid.org/0000-0003-0365-9073

Piyush Chaudhary: http://orcid.org/0009-0005-7839-3638

Kenneth M Ng: http://orcid.org/0009-0006-7898-7994

Daphne Superville: http://orcid.org/0000-0002-8317-8431

Jadyn Leek: http://orcid.org/0009-0001-8346-2934

Joshua Tay: http://orcid.org/0009-0004-0626-8662

Corinna Martinez Luna: http://orcid.org/0009-0006-9299-5106

Maria Gonzalez: http://orcid.org/0000-0002-0701-0645

Eric Smith: http://orcid.org/0000-0002-7510-5603

Dekker C Deacon: http://orcid.org/0000-0002-4160-5730

Allie Grossmann: http://orcid.org/0000-0002-7665-1403

Melissa Q Reeves: http://orcid.org/0000-0002-4886-5164

## Conflict of Interest

The authors state no conflict of interest

## Supplementary Materials

Supplementary Table 1 Full list of mutations in CIT cell lines. Comprehensive list of all mutations identified in each CIT cell line through whole exome sequencing, including chromosome and position coordinates for each mutation corresponding to the mm 10 mouse genome build.
